# The role of critical thinking skills and learning styles of university students in their academic performance

**Published:** 2014-07

**Authors:** ZOHRE GHAZIVAKILI, ROOHANGIZ NOROUZI NIA, FARIDE PANAHI, MEHRDAD KARIMI, HAYEDE GHOLSORKHI, ZARRIN AHMADI

**Affiliations:** 1 Emergency medical services department, Paramedical school, Alborz University of Medical Sciences, Karaj, Iran;; 2 Educational Development Center, Alborz University of Medical Sciences, Karaj, Iran;; 3 Nursing and midwifery school, Shahid Beheshti University of Medical Sciences, Tehran, Iran;; 4 Department of Epidemiology and Biostatistics, Public Health School, Tehran, Iran;; 5 Medical school, Alborz University of Medical Sciences, Karaj, Iran;; 6 Amirkabir University of Technology(Polytechnic), Tehran, Iran

**Keywords:** Learning, Performance, Student

## Abstract

**Introduction:** The Current world needs people who have a lot of different abilities such as cognition and application of different ways of thinking, research, problem solving, critical thinking skills and creativity. In addition to critical thinking, learning styles is another key factor which has an essential role in the process of problem solving. This study aimed to determine the relationship between learning styles and critical thinking of students and their academic performance in Alborz University of Medical Science.

**Methods:** This cross-correlation study was performed in 2012, on 216 students of Alborz University who were selected randomly by the stratified random sampling. The data was obtained via a three-part questionnaire included demographic data, Kolb standardized questionnaire of learning style and California critical thinking standardized questionnaire. The academic performance of the students was extracted by the school records. The validity of the instruments was determined in terms of content validity, and the reliability was gained through internal consistency methods. Cronbach's alpha coefficient was found to be 0.78 for the California critical thinking questionnaire. The Chi Square test, Independent t-test, one way ANOVA and Pearson correlation test were used to determine relationship between variables. The Package SPSS14 statistical software was used to analyze data with a significant level of p<0.05.

**Results:** Our findings indicated the significant difference of mean score in four learning style, suggesting university students with convergent learning style have better performance than other groups. Also learning style had a relationship with age, gender, field of study, semester and job. The results about the critical thinking of the students showed that the mean of deductive reasoning and evaluation skills were higher than that of other skills and analytical skills had the lowest mean and there was a positive significant relationship between the students’ performance with inferential skill and the total score of critical thinking skills (p<0.05). Furthermore, evaluation skills and deductive reasoning had significant relationship. On the other hand, the mean total score of critical thinking had significant difference between different learning styles.

**Conclusion:** The results of this study showed that the learning styles, critical thinking and academic performance are significantly associated with one another. Considering the growing importance of critical thinking in enhancing the professional competence of individuals, it's recommended to use teaching methods consistent with the learning style because it would be more effective in this context.

## Introduction

The current world needs people with a lot of capabilities such as understanding and using different ways of thinking, research, problem solving, critical thinking and creativity. Critical thinking is one of the aspects of thinking that has been accepted as a way to overcome the difficulties and to facilitate the access to information in life (1).

To Watson and Glizer, critical thinking is a combination of knowledge, attitude, and performance of every individual. They also believe that there are some skills of critical thinking such as perception, assumption recognition deduction, interpretation and evaluation of logical reasoning. They argue that the ability of critical thinking, processing and evaluation of previous information with new information result from inductive and deductive reasoning of solving problems. Watson and Glizer definition of critical thinking has been the basis of critical thinking tests that are widely used to measure the critical thinking today (2).

World Federation for Medical Education has considered critical thinking one of the medical training standards so that in accredited colleges this subject is one of the key points. In fact, one of the criteria for the accreditation of a learning institute is the measurement of critical thinking in its students (3).

In addition to critical thinking, learning style, i.e. the information processing method, of the learners, is an important key factor that has a major role in problem solving. According to David Kolb’s theory, learning is a four-step process that includes concrete experience, reflective observation, abstract conceptualization and active experimentation. This position represents two dimensions: concrete experience versus abstract thinking, and reflective observation to active experimentation. These dimensions include four learning styles: divergent, convergent, assimilate, and accommodate. According to Kolb and Ferry, the learner needs four different abilities to function efficiently: Learning styles involve several variables such as academic performance of learner, higher education improvement; critical thinking and problem solving (4).

Due to the importance of learning styles and critical thinking in students' academic performance, a large volume of educational research has been devoted to these issues in different countries. Demirhan, Besoluk and Onder (2011) in their study on critical thinking and students’ academic performance from the first semester to two years later have found that contrary to expectations the students’ critical thinking level reduced but the total mean of students’ scores increased. This is due to the fact that the students are likely to increase adaptive behavior with environment and university and reduce the stress during their education (1).

In another study over 330 students in Turkey, the students who had divergent learning style, had lower scores in critical thinking in contrast with students who have accommodator learning style (5).

Also Mahmoud examined the relationship between critical thinking and learning styles of the Bachelor students with their academic performance in 2012. In this study all the nursing students of the university in the semesters four, six and eight were studied. The results did not show any significant relationship between critical thinking and learning styles of nursing students with their academic performance (6).

Another research by Nasrabadi in 2012 showed a positive relationship between critical thinking attitudes and student's academic achievement. The results showed that there was a significant difference between the levels of critical thinking of assimilating and converge styles. Also converging, diverging, assimilating and accommodating styles had the highest level of critical thinking, respectively (4). Among other studies we can refer to Sharma’s study in 2011 whose results suggested a relationship between the academic performance and learning styles (7).

Today university students should not only think but also should think differently and should not only remember the knowledge in their mind but also should research the best learning style among different learning styles. Therefore, the study on the topic of how the students think and how they learn has received great emphasis in recent years. In this regard, with the importance of the subject, researchers attempted to doa research in this area to determine the relationship between critical thinking and learning styles with academic performance of the students at Alborz University of Medical Sciences. 

## Methods

This study is a descriptive-analytic, cross sectional study and investigates the relationship between critical thinking and learning styles with students’ academic performance of Alborz University of Medical Science in 2012. After approval and permission from university’s authorities and in coordination with official faculties, the critical thinking and learning styles questionnaire was given to the undergraduate students in associate degree, bachelor, medicine (second semester and after that). The total number of participants in the study was 216 students with different majors such as medical, nursing and midwifery, and health and medical emergency students. The tool to collect the data was a two-part questionnaire of Kolb's learning styles and California's critical thinking skills test (form B). The Kolb's questionnaire has two parts. The first part asks for demographic information and the second part includes 12 multiple choice questions. The participants respond to the questions with regard to how they learn, and the scores of respondents are ranked from 1 to 4 in which 4 is most consistent with the participants’ learning style 3 to some extent, 2 poorly consistent and 1 not consistent To find the participants’ learning styles, the first choice of all 12 questions were added together and this was repeated for other choices. Thus, four total scores for the four learning styles were obtained, the first for concrete experience learning style, the second for reflective observation of learning style, the third for abstract conceptualization learning style and the forth for active experimentation learning style. The highest score determined the learning style of the participant. The California critical thinking skills test (form B) includes 34 multiple choice questions with one correct answer in five different areas of critical thinking skills, including evaluation, inference, analysis, inductive reasoning and deductive reasoning. The answering time was 45 minutes and the final score is 34 and the achieved score in each section of the test varies from 0 to 16. In the evaluation section, the maximum point is 14, in analysis section 9, in inference section 11, in inductive reasoning 16 and in deductive reasoning the maximum point was 14. So there were 6 scores for each participant, which included a critical thinking total score and 5 score for critical thinking skills. Dehghani, Jafari Sani, Pakmehr and Malekzadeh found that the reliability of the questionnaire was 78% in a research. In the study of Khalili et al., the confidence coefficient was 62% and construct validity of all subscales with positive and high correlation were reported between 60%-65%. So this test was reliable for the research. Collecting the information was conducted in two stages. In the first stage, the questionnaires were given to the students and the objectives and importance of the research were mentioned. In the next stage, the students' academic performance was reviewed. After data collection, the data were coded and analyzed, using the SPSS 14 ( SPSS Inc, Chicago, IL, USA) software. To describe the data, descriptive statistics were used such as mean and standard deviation for continues variables and frequency for qualitative variables. Chi Square test, Independent t-test, one way ANOVA and Pearson correlation test were used to determine the relationship between variables at a significant level of p<0.05.

**Research hypothesis**

1. There is a relationship between Alborz University of Medical Sciences students’ learning styles and their demographic information. 
2. There is a relationship between Alborz University of Medical Sciences students’ critical thinking and their demographic information. 
3. There is a relationship between Alborz University of Medical Sciences students’ academic performance and their demographic information. 
4. There is a relationship between Alborz University of Medical Sciences students’ learning styles and their academic performance. 
5. There is a relationship between Alborz University of Medical Sciences students’ learning styles and their critical thinking. 

## Results

225 questionnaires were distributed of which 216 were completely responded (96%). The age range of the participants was from 16 to 45 with the mean age of (22.44±3.7). 52.8% of participants (n=114) were female, 83.3% (n=180) were single, 30.1% of participants’ (n=65) major was pediatric anesthesiology of OR, 35.2% of participants (n=76) were in fourth semester, 74.5% (n=161) were unemployed and 48.6 % (n=105) had Persian ethnicity.

The range of participants’ average grade points, which were considered as their academic performance, were from 12.51 to 19.07 with a mean of (16.75±1.3). According to Kolbs' pattern, 42.7% (n=85) had the convergent learning style (the maximum percentage) followed by 33.2 % (n= 66) with the assimilating style and only 9.5%, (n= 19) with the accommodating style (the minimum percentage).

Among the 5 critical thinking skills, the maximum mean score belonged to deductive reasoning skill (3.38±1.58) and the minimum mean score belonged to analysis skill (1.67±1.08).

Table 1 shows the frequency distribution and demographic variables and the academic performance of the students. According to the Chi-square (Χ^2^) p-value, there was a significant relationship between gender and learning style (p=0.032), so that nearly 50 percent of males had the assimilating learning style and nearly 52 percent of the females had the convergent learning style.

The relationship between employment, major and semester of studying with the learning style was significant at a p-value of 0.049, 0.006, 0.009 and 0.001, respectively. The mean and standard deviation of age and students' academic performance in the four learning styles are reported in Table 1.

Using the one way analysis of variance (One way ANOVA) and comparing the mean age of four groups, we found a significant relation between age and academic performance with learning style (p=0.049).

The students with convergent learning style had a better academic performance than those with other learning styles and in the performance of those with the assimilating learning style the weakest.

**Table 1:** The relationship between demographic variable and student’s academic performance with learning styles

**Variable**	**Stages**	**Learning styles**				**p**
		**Divergent**	**Accommodate**	**Convergent**	**Assimilate**	
**Gender**	Male	14 (15.1)	10 (10.8)	30 (23)	39 (41.9)	0.032
	Female	15 (1.2)	9.58	55 (51.9)	27 (25.5)	
**Marriage state**	Single	24 (13.9)	15 (8.7)	77 (44.5)	57 (32.9)	0.470
	Married	5 (19.2)	4 (15.4)	8 (30.8)	9 (34.6)	
**Employment**	Unemployment	26 (17.1)	9 (5.9)	67 (44.1)	50 (32.9)	0.006
	Employed	3 (9.1)	8 (24.2)	10 (30.3)	12 (36.4)	
**Major**	Health	4 (6.9)	4 (6.9)	30 (51.7)	20 (34.5)	0.009
	Nursing, Midwifery	5 (11.6)	1 (2.3)	22 (51.2)	15 (34.9)	
	Anesthesiology/OR	12 (19.4)	11 (7.7)	25 (40.3)	14 (22.6)	
	Medical emergency	8 (22.2)	3 (8.3)	8 (22.2)	17 (47.2)	
**Semester**	Second	2 (4.7)	2 (4.7)	19 (44.2)	20 (46.5)	0.001
	Third	8 (14.8)	3 (5.6)	27 (50.0)	16 (29.6)	
	Fourth	11 (16.7)	14 (6.6)	29 (43.9)	22 (33.3)	
	Fifth and later	6 (18.8)	10 (31.3)	8 (25.0)	17 (25.0)	
**Ethnicity**	Persian	14 (14.6)	12 (12.8)	46 (47.9)	24 (25.0)	0.130
	Turk	9 (15.5)	5 (8.6)	26 (44.8)	18 (31.0)	
	Kurd	3 (10.7)	2 (7.1)	9 (32.1)	14 (50.0)	
	Other	3 (17.6)	0 (0.0)	4 (23.5)	10 (58.8)	
** Performance(Mean±SD) **	1.50±21.7	23.20±3.10	22.07±2.65	44.22±3.73	0.114
	16.66±1.19	16.27±1.09	17.07±1.13	16.50±1.26	0.040

Table 2 shows the relationship between the total score of critical thinking skills and each of the demographic variables and academic performance. The results of the t-test and one way ANOVA variance analysis are reported to investigate the relationship between each variable with skills below the mean standard deviation.

Based on the t-test and ANOVA, p-value of t and F, the mean of total score of critical thinking skills had only significant relationship with students’ major (p=0.020). Also a significant relationship was found between the major of students and gender with inference skill; semester of study with deductive reasoning skill, and ethnicity with 2 skills of inference and deductive reasoning (p<0.05). 

Also regarding the relationship between age and the student academic performance with each of the critical thinking skills, the Pearson correlation coefficient results indicated a significant positive relationship but a negative relationship between age and analysis skill, i.e. with the increase of age, the score of analysis skill was reduced (p<0.05). Academic performance of the students had a direct significant relationship with critical thinking total score and inference skill; the more the score, the better the academic performance of students (p<0.05).

**Table 2:** Relationships between CCT Skills and demographic variables Using t-test and ANOVA. Pearson Correlation coefficient between age and Student's performance with CCT Skills was reported

**Variable**	**Categories**	**Critical thinking skills**					
		**Assessment** **Mean±SD**	**Analysis** **Mean±SD**	**Inference** **Mean±SD**	**Reasoning** **Mean±SD**	**Conclusion** **Mean±SD**	
**Sex**	**Male**	3.20±1.71	1.68±1.11	2.03±1.15	3.28±1.56	2.94±1.50	
	**Female**	3.18±1.39	1.66±1.05	2.44±1.28	3.47±1.61	3.23±1.57	
	**p**	0.927	0.926	0.021	0.406	0.198	
**Marital Status**	**Single**	3.27±1.59	1.72±1.05	2.21±1.19	3.40±1.64	3.15±1.52	
	**Married**	2.81±1.30	1.42±1.20	2.40±1.43	3.28±1.30	2.81±1.63	
	**p**	0.088	0.157	0.418	0.690	0.256	
**Employment**	**Not occupying**	3.21±1.53	1.66±1.06	2.23±1.22	3.41±1.65	3.07±1.56	
	**Occupying**	3.15±1.63	1.70±1.14	2.27±1.29	3.29±1.39	3.17±1.51	
	**p**	0.836	0.841	0.835	0.642	0.700	
**Major**	**Sanitation**	2.86±1.58	1.63±1.03	1.93±1.24	2.88±1.57	2.97±1.45	
	**Nursing & Midwifery**	3.15±1.31	1.42±1.03	2.38±1.15	3.44±1.57	2.97±1.52	
	**Paramedic**	3.42±1.69	1.86±1.11	2.53±1.21	3.65±1.64	3.48±1.63	
	**EMT**	3.23±1.51	1.71±1.13	1.94±1.27	3.42±1.44	2.73±1.44	
	**p**	0.332	0.211	0.024	0.100	0.083	
**Semester**	**Second**	2.89±1.85	1.81±1.19	2.53±1.35	3.28±1.87	3.27±1.50	
	**Third**	3.42±1.34	1.49±1.06	2.35±1.38	3.46±1.58	3.29±1.57	
	**Fourth**	3.03±1.53	1.63±0.90	1.98±1.06	3.27±1.47	2.67±1.50	
	**Fifth & higher**	3.54±1.48	2.03±1.19	2.26±1.13	3.72±1.52	3.39±1.57	
	**p**	0.181	0.117	0.130	0.568	0.049	
**Ethnicity**	**Persian**	3.27±1.60	1.55±1.07	2.49±1.25	3.44±1.71	2.38±1.58	
	**Turk**	3.16±1.52	1.75±1.09	2.00±1.23	3.32±1.52	2.86±1.48	
	**Kurd**	3.18±1.56	1.89±1.10	1.92±1.08	3.28±1.32	2.92±1.41	
	**Others**	2.87±1.40	1.68±1.07	2.25±1.18	3.43±1.59	2.56±1.59	
	**p**	0.816	0.438	0.045	0.953	0.047	
**Pearson correlation coefficient** ** **							
**Age (Correlation)**		-0.008	-0.041	0.059	0.023	-0.056	-0.070
**P**		0.285	0.041	0.425	0.761	0.448	0.36
**Performance (Correlation)**		-0.003	0.075	0.158	0.095	0.028	0. 149
**P**		0.97	0.29	0.027	0.194	0.698	0.044

* Significant in surface 0.05

** Significant in surface 0.01

Table 3 shows the mean and standard deviation of learning styles score in the 4 groups of learning style. Using ANOVA one way ANOVA, the relationship between learning style and critical thinking skills and the comparison of the mean score for each skill in four styles are reported in the last column of the Table 3.

Based on the p-value of ANOVA, the mean of evaluation skill and inductive reasoning skill had a significant difference and the relationship between these two skills with learning style was significant (p<0.05). Also the mean of critical thinking’s total score was significantly different in the four groups and the relationship between total score with learning style was significant, too (p<0.05).

**Table 3:** The Relationship between critical thinking styles with learning styles

**CCT Skills**	**Learning styles**				**p**
	**Divergent**	**Accommodate**	**Convergent**	**Assimilate**	
	**Mean±SD**	**Mean±SD**	**Mean±SD**	**Mean±SD**	
**Assessment**	3.40±1.29	3.66±1.57	3.29±1.59	2.70±1.61	0.045
**Analysis**	1.91±1.24	1.88±1.07	1.69±1.11	1.43±0.96	0.185
**Deduction**	1.91±1.24	2.33±0.90	2.29±1.25	2.25±1.31	0.594
**Inductive reasoning**	3.59±1.59	3.83±1.50	3.53±1.61	2.81±1.59	0.028
**Deductive reasoning**	2.83±1.52	3.44±1.75	3.03±1.53	3.13±1.49	0.625
**CCT total score **	7.33±2.10	7.88±2.56	7.30±2.44	6.41±2.52	0.032

**Figure 1:**
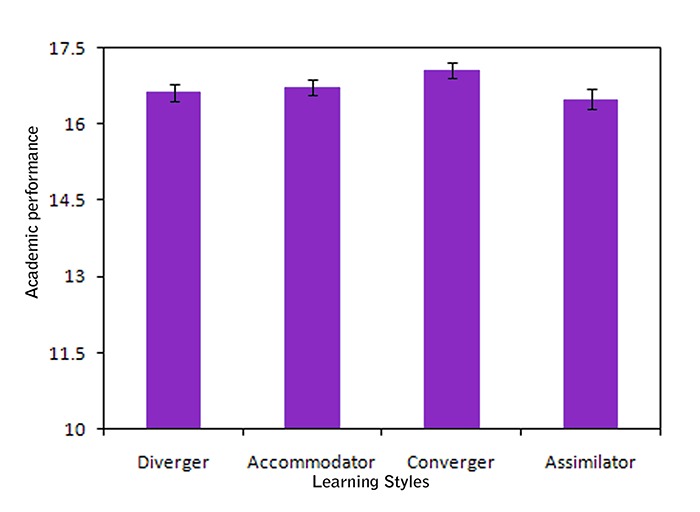
The mean and confidence interval of university students’ performance in four learning  styles

**Figure 2:**
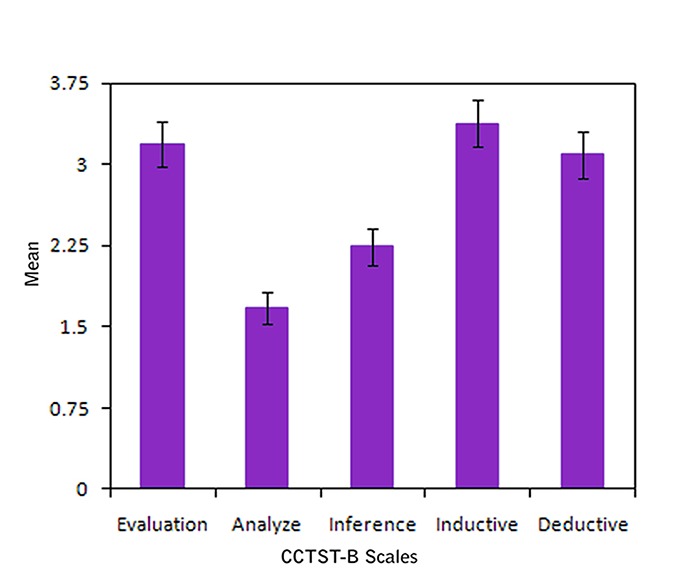
The mean and confidene interval of critical thinking skills

## Discussion

The study findings showed that the popular learning style among the students was the convergent style followed by the assimilating style which is consistent with Kolb's theory stating that medical science students usually have this learning style (8). This result was consistent with the results of other studies (9, 10). In Yenice's study in which the student of training teacher were the target of the project, the most frequent learning styles were divergent and assimilating styles and these differences originate from the different target group of study in 2012 (11).

This study showed a significant relationship between learning style and gender, age, semester and employment. Meyari et al. did not find any significant relationship between learning style, age and gender of the freshman but for the fifth semester students, a significant relationship with age and gender was found (10). Also in Yenice's study, no relationship with learning style, gender, semester and age was found.

Furthermore, in the first semester divergent style, in the second semester assimilating style and in the third and fourth semester divergent style were accounted for the highest percentage. Also in the group age of 17-20 years the assimilating style and the age of 21-24 years the divergent style were dominant styles (11).

In the present study, it was found a significant positive relationship between convergent learning style and academic performance. Also in the study of Pooladi et al. the majority of the students had convergent style and they also found a significant relationship between learning style, total mean score and the mean of practical courses (12). Nasrabadi et al. found that students with the highest achievement were those with convergent style with a significant difference with those with divergent style (4). But the results are inconsistent to Meyari et al.’s (10).

In this study, the obtained mean score from the critical thinking questionnaire was (7.15±2.41) that was compared with that in the study of Khalili and Hoseinzadeh which was to validate and make reliable the critical thinking skills questionnaire of California (form B) in the Iranian nursing students; the mean of total score was about the 11th percentile of this study (13).

In other words, the computed score for critical thinking of the students participating was lower than 11 score that is in the 50th percentile and of course is lower than normal range.

Hariri and Bagherinezhad had shown that the computed score for Bachelor and Master students of Health faculty was also lower than the norm in Iran (14). Also Mayer and Dayer came to a similar conclusion in critical thinking skill in the Agricultural university of Florida’s students in 2006 (15).

But in Gharib et al.’s study, the total score of critical thinking test among the freshman and senior of Health-care management was in normal range (16). Wangensteen et al., found that the critical thinking skills of the newest graduate nursing students were relatively high in Sweden in 2010 (17).

In this study, students of all levels (Associate, Bachelor and PhD) with various fields of study participated but other studies have been limited to certain graduate courses that may explain the differences in levels of special critical thinking skills score in this study. In this study we found a significant relationship between total score of critical thinking and major of the students. This result is consistent with Serin et al. (18).

It was found a significant relationship between major of participants, gender and inference skill, semester and deductive reasoning skill, ethnicity and both inference and deductive reasoning skills.

In the Yenice's study significant relationship between critical thinking, group of age, gender and semester was seen (11). In Wangensteen et al.’s (17) study in the older age group, the level of critical thinking score increased. In Serin et al.’s (18) study the level of communication skills in girls was better than that in boys. And also a significant relationship was found between critical thinking and academic semester, but in Mayer and Dayer’s study no significant relationship between critical thinking levels and gender was found (4,15).

The results also showed that the total score of critical thinking and analytical skills of students and their performance had a significant relationship. Nasrabady et al.’s study also showed that there was a positive relationship between critical thinking reflection attitude and academic achievement (4). This is contradictory with what Demirhan, Bosluk and Ander found (6, 15).

The results of the relationship between learning style and critical thinking indicated that the relationship between evaluation and inductive reasoning was significant to learning style (p<0.05). The relationship of critical thinking total score with learning style was also significant (p<0.05). Thus the total score for those with the conforming style of critical skills was more than that with other styles. But in the subgroup of inference skills, those with the convergent style had a higher mean than those with other styles.

Yenice found a negative relationship between critical thinking score and divergent learning style and a positive relation between critical thinking score and accommodating style (11).

Siriopoulos and Pomonis in their study compared the learning style and critical thinking skills of students in two phases: at the beginning and end of education and came to this conclusion that the learning style of students changed in the second phase.

For example, the divergent, convergent and accommodating styles languished and the assimilating style (combination of abstract thinking and reflective observation) was noticeably strengthened. However, those with converging learning style had higher levels of critical thinking.

The level of students’ critical thinking was lower in all international standards styles. Perhaps it was because of widely used teacher-centered teaching methods (lectures) in that university (19).

The results in the study of Nasrabady et al. showed that there was a significant difference between the level of learners’ critical thinking and divergent and assimilating styles (4).

Those with converging, diverging, assimilating and accommodating styles had the highest level of critical thinking, respectively.

Also there was a positive significant relationship between the reflective observation method and critical thinking and also a negative significant relationship between the abstract conceptualization method and critical thinking (4). But in another study that Mahmud has done in 2012, he did not find any significant relationship between learning style, critical thinking and students’ performance (6).

## Conclusion

The results of this study showed that the students’ critical thinking skills of this university aren't acceptable. Also learning styles, critical thinking and academic performance have significant relationship with each other. Due to the important role of critical thinking in enhancing professional competence, it is recommend using teaching methods which are consistent with the learning styles. 
